# Role of space station instruments for improving tropical carbon flux estimates using atmospheric data

**DOI:** 10.1038/s41526-022-00231-6

**Published:** 2022-11-20

**Authors:** Paul I. Palmer, A. Jerome P. Woodwark, Douglas P. Finch, Thomas E. Taylor, André Butz, Johanna Tamminen, Hartmut Bösch, Annmarie Eldering, Sebastien Vincent-Bonnieu

**Affiliations:** 1 School of GeoSciences, University of Edinburgh, Edinburgh, UK; 2 National Centre for Earth Observation, University of Edinburgh, Edinburgh, UK; 3 Cooperative Institute for Research in the Atmosphere, Colorado State University, Fort Collins, CO USA; 4 Institute of Environmental Physics, Heidelberg University, Heidelberg, Germany; 5 Finnish Meteorological Institute, Helsinki, Finland; 6 National Centre for Earth Observation, University of Leicester, Leicester, UK; 7 Earth Observation Science, School of Physics and Astronomy, University of Leicester, Leicester, UK; 8 Jet Propulsion Laboratory, California Institute of Technology, Pasadena, CA USA; 9 Directorate of Human and Robotic Exploration Programmes, European Space Agency – ESTEC, Noordwijk-ZH, The Netherlands

**Keywords:** Environmental monitoring, Environmental monitoring, Biogeochemistry

## Abstract

The tropics is the nexus for many of the remaining gaps in our knowledge of environmental science, including the carbon cycle and atmospheric chemistry, with dire consequences for our ability to describe the Earth system response to a warming world. Difficulties associated with accessibility, coordinated funding models and economic instabilities preclude the establishment of a dense pan-tropical ground-based atmospheric measurement network that would otherwise help to describe the evolving state of tropical ecosystems and the associated biosphere-atmosphere fluxes on decadal timescales. The growing number of relevant sensors aboard sun-synchronous polar orbiters provide invaluable information over the remote tropics, but a large fraction of the data collected along their orbits is from higher latitudes. The International Space Station (ISS), which is in a low-inclination, precessing orbit, has already demonstrated value as a proving ground for Earth observing atmospheric sensors and as a testbed for new technology. Because low-inclination orbits spend more time collecting data over the tropics, we argue that the ISS and its successors, offer key opportunities to host new Earth-observing atmospheric sensors that can lead to a step change in our understanding of tropical carbon fluxes.

## Introduction

The tropics, loosely defined here as the area between the Tropic of Cancer and the Tropic of Capricorn, encompasses parts of central and South America; parts of West, East, Central and Southern Africa; Madagascar; parts of India; mainland and maritime Southeast Asia; and northern parts of Australia. The tropics represents ~30% of Earth’s land mass, including most of Earth’s rainforests that contribute ~60% of global land-based gross primary production^1^, wetland ecosystems that explain more than ~80% of the observed global atmospheric methane growth rate since 2010 (ref. ^2^), and ~80% of global terrestrial isoprene emissions^3,4^. They are a foci for wildfires and managed fires^5^, and 40% of the world population^6^. Projections of population growth from now until the end of the 21st century are driven by low latitude countries mostly in sub-Saharan Africa and Asia^7^.

Increased warming due to rising levels of atmospheric greenhouse gases could, in the worst-case scenario, result in the eventual destabilization of vast above-ground and/or below-ground carbon stores^8–10^ leading to elevated carbon emissions that would accelerate warming resulting in massive societal impacts. One loci for such Earth system bifurcations is the tropics^11^. Crucially, we do not currently have sufficient measurements over the tropics to understand how natural ecosystems are responding to contemporary changes in climate, which provide a guide to how they might respond to future changes in climate. Poor measurement coverage over the tropics is exacerbated by extensive, seasonal (and diurnal) cloud coverage^12^ (wet seasons) and elevated aerosol loading (e.g., due to burning in dry seasons). Here, we focus on science questions about tropical carbon fluxes that can be addressed by atmospheric measurements of CO_2_, methane and reactive chemistry trace gases collected by instruments installed on space stations, taking advantage of their low inclination orbits, and put them into context of data collected by polar-orbiting satellites.

## Current and near-future Earth observing measurement capabilities

We summarise the current and near-future atmospheric measurement capabilities that are relevant for quantifying natural carbon fluxes and anthropogenic carbon emissions, including instruments on the ISS.

### Current Earth observing capabilities for the tropics

Most science-driven Earth Observing satellites are in a sun-synchronous (low Earth) orbit (typically 600–800 km above Earth) which is a particular type of polar orbit where the orbital plane remains in a fixed position in the Earth-Sun reference frame: on-board nadir-viewing instruments then observe points on the Earth’s surface at the same local time of day and night, separated by 12 h. The ground spacing between successive orbits of a spacecraft in a sun-synchronous orbit is largest at low latitudes. Across-track sampling (e.g., scanning or push-broom configurations) helps minimise these gaps; measurement gaps can also be addressed by satellite constellations. Geostationary orbits (35,786 km above the equator with zero inclination and eccentricity) match the rotational period of the Earth so that fixed-pointing, nadir-viewing instruments stare at the same location on the surface, viewing a disc that can encompass the entire projected globe. For these orbits, the measurement quality degrades at higher viewing angles towards the disc edges, associated with longer atmospheric light paths (resulting in poorer horizontal resolution) for which our path-integrated knowledge of the aerosol optical properties becomes progressively poorer. These orbits have the advantage of observing the same region throughout the day, although this might be limited to daytime, cloud-free scenes depending on the type of deployed instruments e.g., whether they collect reflected sunlight or carry their own laser light source or measure the thermal infrared emission of the Earth. Geostationary missions are expensive due to their high orbital altitudes (associated with a costly launch) and competition for a limited number of satellite positions. Higher spatial resolution geostationary instruments typically view only a portion of the tropics, although there is scope to build virtual constellations to improve coverage^13–16^.

A wide range of sun-synchronous satellite instruments currently collect data that inform our understanding of tropical carbon fluxes and atmospheric chemistry^17^. Instruments that measure short-wave infrared (SWIR) wavelengths are more sensitive to changes in atmospheric CO_2_ and methane in the lower troposphere close to the surface fluxes. These include the Japanese Greenhouse gases Observing SATellite, GOSAT (CO_2_ and methane, launched in 2009 (ref. ^18^)), and its follow-on GOSAT-2 (CO_2_, methane, and CO, launched in 2018 (ref. ^19^)); NASA OCO-2 (CO_2_, launched in 2014 (refs. ^20,21^)); the Chinese TanSAT mission (CO_2_, launched in 2016 (refs. ^22,23^)); and the TROPOspheric Monitoring Instrument, TROPOMI (methane and atmospheric chemistry, launched in 2017 (ref. ^24^)) aboard the Copernicus Sentinel-5 Precursor satellite. Instruments that observe complementary thermal IR wavelengths, such as the Infrared Atmospheric Sounding Interferometer, IASI (methane and atmospheric chemistry, launched on MetOp satellites 2006, 2012, and 2018 (ref. ^25^)) and the NASA Atmospheric Infrared Sounder, AIRS (CO_2_, methane, CO, launched in 2002 (ref. ^26^)), are more sensitive to changes of CO_2_ and methane in the mid-troposphere. With the exception of IASI, these instruments are science-driven explorer missions that were launched to demonstrate technological advances and to showcase the scientific advances supported by the resulting data. Data from OCO-2, GOSAT and TROPOMI, for example, have revealed new insights into the tropical carbon cycle^2,27–32^, with TROPOMI in particular improving our understanding of tropical methane fluxes by virtue of its unprecedented spatial resolution and better daily coverage^31,33–35^. The transition to operational data streams occurred more than a decade ago with hyperspectral^25,36^ and solar backscatter ultraviolet^37,38^ instruments installed on US, Japanese, and European weather satellites, building on substantial heritage^39–41^.

### Near-future Earth observing capabilities for the tropics

There is now a move towards operational delivery of CO_2_ and methane data, motivated by the need to increase the measurement and verification support (MVS) capacity to support the Paris Agreement. Arguably, the biggest change will result from data delivered by the upcoming Copernicus CO_2_ Monitoring Mission (CO2M)^42^ that is due for launch in 2025. CO2M will have an equatorial local overpass time of 11:30, focused on quantifying anthropogenic emissions of CO_2_ and methane and will form part of the European MVS measurement and verification support capacity^43^. CO2M will likely consist of 2–3 satellites, each with a push-broom imaging spectrometer that has an across-track swath of ~250 km with a spatial resolution of 4 km^2^. Each satellite has an 11-day repeat cycle so that global coverage will be achieved in approximately four to six days with three and two satellites, respectively. As a result, geographical locations will be sampled at most five to eight times per month, depending on clouds and atmospheric aerosols, and typically much less at tropical latitudes where clouds are prevalent. Each spectrometer will measure four spectral bands that cover the SWIR wavelengths necessary to quantify columns of CO_2_ and methane and the visible wavelengths necessary to quantify NO_2_ columns, which will be used as a proxy for combustion. Each satellite will also include a multi-angle polarimeter for aerosol measurements and a cloud imager.

Column observations of CO_2_ from CO2M have an estimated precision requirement (1-sigma) of <0.7 ppm^42^. This value is comparable to observed precision values 0.8 ppm (land) and 0.5 ppm (ocean) from OCO-2, which has slightly smaller ~3 km^2^ footprints^44^. CO2M will provide a step change in the volume of available CO_2_ and methane data, building on our current capabilities. However, CO2M will not address all carbon cycle questions. By virtue of its primary objective, it will not be coincident with any ecosystem or atmospheric chemistry measurements (with the exception of NO_2_) that as we discuss later enhances our ability to interpret the CO_2_ and methane data. However, it will include retrievals of solar induced fluorescence (SIF) that is used as a proxy for gross primary production^45^, accounting for changes in sun angle that affect the radiative transfer in the canopy^46^. Observing at the same local time of day may cause biases in tropical data coverage, impacted by diurnal and seasonal changes in clouds and aerosol loading. CO2M will be less sensitive to carbon hotspot emissions on scales <2 km compared with the next generation of imaging spectrometers that have finer spatial resolution^47,48^.

Despite massive advances in the number and quality of sun-synchronous satellite observations of the carbon cycle and atmospheric chemistry over the last decade, there remain gaps in our scientific understanding that can be addressed using Earth observing instruments installed on the ISS and its successor space stations.

### Earth observing capabilities on the ISS

**Fig. 1:**
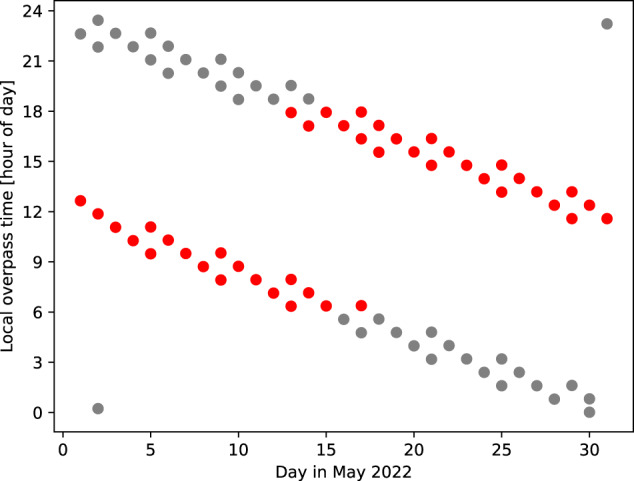
Local overpass time of the ISS over Kinshasa (4.4246°S, 15.1847°E), Democratic Republic of the Congo, during May 2022. Red and grey dots denote overpasses that are during the sunlit day and night-time, respectively. Here an overpass is defined as times when the ISS is visible at an elevation of more than 10 degrees above the horizon. We have not considered cloud coverage or the maximum elevation angle above the local horizon: both factors will influence the quality of measurements collected. Points in the bottom left and top right corners mark the end of the preceding and the start of the following repeated overpass changes, respectively. Data are taken from heavens-above.com. Figure drawn by P.I.P. with contributions from A.J.P.W.

The ISS is a joint project between NASA, Roscosmos, JAXA, ESA, and the CSA. It is in a low Earth orbit inclined at 51.6^o^, completing 15.7 orbits per day. As a result of its orbit, the ISS precesses around the Earth between latitudes of ±51.6^o^ (Fig. 1). The mean orbital altitude is about 400 km (330–425 km) that is reduced by 2 km/month due to atmospheric drag and therefore requires periodic orbital boosts to maintain a minimum altitude above Earth. The ISS includes multiple pressurised and external payloads sites that have hosted microgravity and space environment research experiments. The ISS has only been recently considered as a platform to host Earth observing instruments of which five instruments are associated with tropical ecosystems (Table 1), including one relevant to atmospheric remote sensing (OCO-3). Most of these Earth observing instruments have been hosted on the external Japanese Experiment Module (JEM, nicknamed Kibō). ESA has not yet exploited the opportunity to use the ISS as a platform for EO instruments. While these are standalone instruments that target key science questions, many of them are addressing different aspects of a broader scientific challenge (e.g., the links between water, phenology, and carbon uptake) so there is tremendous value in combining their data^49,50^.

**Table 1:** Earth observing instrument aboard the ISS relevant for studying tropical ecosystems.

Instrument name/responsible agency	Primary objective	Duration	Ref.
Orbiting Carbon Observatory (OCO-3)/NASA	Measure atmospheric CO_2_ and SIF to investigate carbon cycle.	May 2019–2023^a^	^54,55^
DLR Earth Sensing Imaging Spectrometer (DESIS)/DLR	Hyperspectral measurements of surface reflectance.	June 2018–2023^a^	^151,152^
ECOsystem Spaceborne Thermal Radiometer Experiment on Space Station (ECOSTRESS)/NASA	Mapping evapotranspiration to improve understanding of water usage of plants	June 2018–2023^a^	^153^
Global Ecosystem Dynamics Investigation (GEDI)/NASA	Characterise the effects of changing climate and land-use on ecosystem structure and dynamics.	December 2018–2023^a^	^154^
Hyperspectral Imager Suite (HISUI)/JAXA	Remote sensing of oil/gas/mineral resource and agriculture, forestry, and coastal issues	December 2019–2024^a^	^155^

^a^Planned dates for disposal at the time of writing, but these may be subject to change in the future.

There are advantages and disadvantages of using the ISS (also generally using an inclined low Earth orbit) as an Earth observing platform to collect observations of greenhouse gases and other trace gases, depending on the measurement being collected. Generally, the inclination of the orbit defines the latitude range over which data can be collected. The ISS orbital inclination of 51.6^o^, driven by constraints associated with Russian launches, is consequently not optimised to observe the tropics. To the authors’ knowledge only three Earth observing missions have used inclined orbits to collect science data, all of which have used orbital inclinations lower than the ISS to focus on tropical precipitation^51,52^ and tropical storms^53^.

The main advantage of an inclined orbit is that a limited set of latitudes is sampled at different local times of day, including during nighttime hours (Fig. 1). For an Earth observing instrument to take full advantage of this it would need to have nightside observing capabilities, i.e., not relying on reflected solar radiation. Even for instruments that do rely on the Sun, there is a clear benefit of discarding some observed scenes in favour of sampling the atmosphere over source regions that include emissions with a diurnal cycle. The main disadvantage of an inclined orbit is the converse argument for its advantage: it samples a limited range of latitudes at different times of day, so the resulting data, especially related to atmospheric composition, are difficult to interpret without a time-dependent computational model that includes a description of emissions and atmospheric chemistry and transport. However, this is also true when interpreting data collected by instruments in sun-synchronous orbits. Global level 3 data products, typically time-averaged orbital data placed on a regular grid, do not reflect a real-life physical picture of the Earth. This is because the same local time on Earth represent different values of universal time. The stability of the ISS platform is also a challenge, but this can be overcome by precision pointing systems such as the one provided on the MUSES platform. There are also programmatic challenges associated with prioritising the installation and operation of Earth observing instruments on a platform primarily intended to study aspects of humans in space. Interruptions in instrument operations due to, for example, entering/exiting vehicles, space walks, station outgassing events, result in gaps in science measurements.

The OCO-3 instrument (Table 1), operating on the ISS since May 2019 (refs. ^54,55^), is already providing CO_2_ measurements across the daytime part of the diurnal cycle. OCO-3 is the first satellite sensor to probe the diurnal cycle of CO_2_ and SIF by virtue of the precessing nature of the ISS orbit. OCO-3, a fundamentally similar instrument to OCO-2, is equipped with an external pointing mirror assembly, which allows fast and accurate control of the observation targeting. The nominal pointing strategy is to collect measurements in glint viewing over large water bodies (to maximise signal to noise ratios) and nadir viewing over land. In addition, OCO-3 collects Target measurements over ground calibration sites and Snapshot Area Maps (SAMs) over regions of interest such as megacities and power plants.

## Science questions relevant to the tropics

We have laid out the scientific questions in no order of importance, but all are relevant to improving the basic scientific understanding of tropical ecosystems and how they will be affected by future climate. First, we focus on land biosphere fluxes of CO_2_ and methane, and emissions of biogenic volatile organic compounds relevant to atmospheric chemistry; and then focus on anthropogenic emissions of air pollutants, CO_2_, and methane. Each topic we discuss is relevant to one or more UN sustainable development goals (SDGs)^56^. Baseline residence times that are necessary for the individual ISS instruments to address these science questions will differ with the data being collected. Rigorous estimates of these residence times require detailed calculations that are outside the scope of this article, but a rule of thumb would be at least 2–3 full seasonal cycles for the natural fluxes, allowing the study of year-to-year variations particularly associated large-scale climate variations, e.g., the El Niño Southern Oscillation.

### Land biosphere fluxes

The tropical land biosphere maintains a large fraction of Earth’s biodiversity (flora and fauna)^57^ that is subject to deforestation and other changes in land use and in climate (e.g. El Niño Southern Oscillation and the Indian Ocean Dipole) that impact ecosystem functioning and consequent atmosphere-biosphere fluxes, and potentially increases the exposure of humans to a range of zoonotic diseases^58^.

#### CO_2_ fluxes

Primary production of tropical ecosystems, converting light into chemical energy thereby drawing in CO_2_ and water from the atmosphere and producing carbohydrates and oxygen, is responsible for absorbing a large fraction of CO_2_ emitted by anthropogenic emissions. Recent analysis of CO_2_ column data collected by the NASA Orbiting Carbon Observatory (OCO-2)^20,21^ and the Japanese GOSAT satellite^18^ have revealed new information about atmosphere-biosphere fluxes that are different from what we expect from process-based models^27,30,59^ and to some extent different from sub-continental scale fluxes inferred from ecological data^60^, and consequently have generated substantial debate in the community^61–63^. In these circumstances, carbon flux estimates are supported by a range of correlative satellite observations of trace gases and land surface properties. A thread running through all science questions associated with tropical ecosystems is the synergistic use of all available data^17,49,50,64^, particularly if satellite observations highlight new results, in order to understand the underlying reasons for changes in surface fluxes. Leading candidates for improving our understanding of CO_2_ fluxes include carbonyl sulfide^65^ and SIF, both of which are proxies for gross primary production; carbon monoxide, formaldehyde (HCHO), and nitrogen dioxide to identify wildfires^30,66–69^; and gravitational anomalies as a proxy for total water storage^30,70,71^. With the exception of gravitational anomalies, the other measurements could, in principle, be accommodated on the ISS to accompany measurements of CO_2_.

Although the varied time-of-day sampling from the ISS provides the prospect for studies of the diurnal carbon cycle, this is not the primary argument for continuing measurements of CO_2_ from the ISS beyond the limited OCO-3 lifetime. Column CO_2_ measurements already represent a superposition of contributions from different geographical regions and from different times^72,73^. An overpass time in early afternoon is most sensitive to the diurnal peak of carbon uptake that generally provides only a weak imprint on the column; interpreting data collected during early/mid-morning overpass is complicated by the covariance between the growing boundary layer and times when the photosynthetic fluxes begin to outcompete respiration fluxes. This formed part of the overall argument for the early afternoon (~13:30) equatorial overpass time of OCO-2 and GOSAT. A key argument for continuing CO_2_ observations from the ISS is that, since the satellite spends a larger fraction of time in the tropics by avoiding the higher latitudes (as with a polar orbiter), the density of measurements within the tropics will necessarily be higher, and as a result increases the quality and resolution of estimated CO_2_ fluxes^74^. This includes an improvement in our ability to partition between ocean and continental fluxes of CO_2_ that respond to climate variations^27,75^. There is keen interest in the information content of the spatially dense OCO-3 SAMs, with a focus on urban hotspots^76,77^, but the majority (~80%) of the OCO-3 data are collected in the nominal land-nadir and ocean-glint viewing modes, spanning latitudes ±51.6^o^ (ref. ^54^). These soundings from the nominal viewing modes support the investigation of natural carbon fluxes, similar to the atmospheric inversion of data collected by OCO-2^29,30,78^. Also, many of the sites selected for SAMs include natural vegetation (especially of interest for SIF), which are often collocated with various ground sensors, and can be used to quantify the associated diffuse CO_2_ fluxes at regional to city-scales.

#### Methane emissions

Large global growth in atmospheric methane concentration since 2007 has evaded a definitive explanation, and a continuation of the record breaking growth rates in 2020 and 2021, both more than 15 ppb/year, may compromise the outcomes of the Paris Agreement^79^. Given concurrent trends towards light isotope signatures from low latitudes for the 13C/12C isotopic ratio it is likely that recent growth in atmospheric methane is influenced substantially by natural sources over the tropics^80,81^. This is broadly consistent with a growing body of work that have identified substantial wetland emissions over the Amazon basin^82^ and over East Africa^28,31,33^. Changes in rainfall, driven by local and remote processes, are predominately responsible for observed changes in the atmospheric methane growth rate in the past decade^2^. Similar to the argument for a CO_2_ instrument, a larger number of clear-sky scenes will improve our ability to detect elevated methane columns due to wetlands emissions.

#### Emissions of biogenic volatile organic compounds

**Fig. 2:**
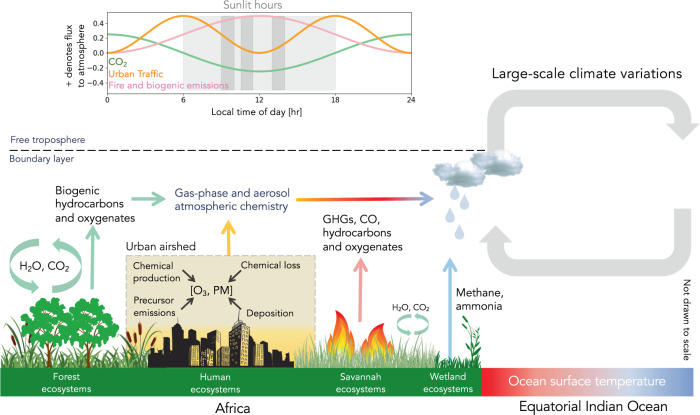
Schematic of some physical and chemical processes that affect the atmospheric distribution of GHGs and atmospheric chemistry over the tropics. The diurnal cycle of emissions over Africa is shown for illustrative purposes. Figure drawn by P.I.P.

Natural vegetation emits hundreds (if not thousands) of compounds that they use to, for example, communicate with other plants, attract pollinators, ward off pests^83^, and to help regulate heat stress^84^. The broad class of these compounds are called volatile organic compounds (VOCs). Broadly speaking, biogenic VOC emissions increase exponentially with warmer temperatures, typically up to 35–44 °C after which emissions plateau, and, for most vegetation, increase with higher levels of photosynthetic active radiation (Fig. 2). These VOC emissions are small compared to net primary production, but they represent a significant source of reduced carbon to the atmosphere^85^ some of which will be oxidised to CO_2_. The importance of VOC emissions lies in their subsequent atmospheric chemistry, with oxidation producing a source of precursors of tropospheric ozone and organic aerosol^86–89^, both relevant for determining Earth’s radiative balance. Isoprene is the dominant non-methane VOC emission to the atmosphere, representing ~500 TgC^3,90^ of which 80% is emitted by tropical ecosystems. Over the Amazon basin, arguably the most observed tropical region, isoprene and reactive biogenic VOCs represent more than 50% of the OH reactivity^91^, with new results suggesting that oxygenated VOCs also play a significant role^92^.

Satellite observations of HCHO have played a key role in understanding tropical isoprene emissions^69,93–96^. HCHO is a high yield product of VOC oxidation^97^ and despite uncertainties in VOC oxidation in photochemical environments with low nitrogen oxides, calculations suggest that observed variations in HCHO are linked to isoprene emissions. There is still much we do not understand about isoprene emissions and its drivers over the tropics^94,98^ and how they will change with future climate^99,100^. Recent studies have showed that isoprene can be retrieved directly from IR spectra measured by a hyperspectral sounder^101–103^, with subsequent analysis revealing a wealth of new knowledge about the seasonal distribution of isoprene emissions across the tropics^103^, including unexpected detections of nighttime isoprene that originate from rapid vertical mixing near sunset, linked to the formation of downwind secondary organic aerosol^102^. Over isoprene hotspots such as the Amazon basin, fine-scale gradients can be resolved at daily resolution from low-Earth orbit measurements^104^. As the volume of data increases, so will our ability to document changes in the distribution and timing of emissions linked to changes in the photochemical environmental and the broader physical climate. There is a strong diurnal cycle of isoprene emissions (Fig. 2) peaking after midday when temperature and light levels are highest, which is not well served using current operational satellites. Installing an IR hyperspectral sounder on the ISS capable of observing isoprene would vastly increase the scope of environmental science and allow scientists to explore atmospheric pollutants during the day and night. This measurement capability would greatly improve our current ability to study the influence of biogenic and pyrogenic emissions on atmospheric chemistry, complementing data collected by polar-orbiting hyperspectral sounders (the Cross-track Infrared Sounder and IASI).

### Anthropogenic emissions

Here we consider anthropogenic emissions to include air pollutants and greenhouse gases. They have different observing requirements that would be served by the ISS and will address different science objectives and SDGs. Aside from their intrinsic value, air pollutants associated with combustion can be used directly to estimate combustion emissions of greenhouse gases or to separate natural and combustion contributions to atmospheric greenhouse gases.

#### Air pollutants

Air pollution is now acknowledged to be the largest environmental stressor on human health^105^, with the effects most acutely felt by those living in the world’s largest cities^106^ where pollutant levels often far exceed World Health Organisation air quality guidelines. There is a pressing need for improved scientific understanding of the emissions and chemistry of reactive trace gases and aerosol to underpin numerical models that can inform and guide global urban development choices over the next two decades. This is particularly relevant to supporting sustainable and healthy urbanisation at low latitudes, i.e., in Africa, Asia, and South America, regions where urbanisation is particularly rapid^107^ and our understanding of emerging air quality challenges is extremely poor, limited to case studies using low-cost sensors or proxies^108–111^. The share of global exports of goods originating from developing countries has been about 45% since 2012 (ref. ^112^), representing a large source of national income but also a significant source of transboundary pollution^113^.

Many of these rapidly growing cities are already encroaching on peri-urban ecosystems associated with emissions from agriculture (including seasonal burning of post-harvest residues^114,115^) and biogenic emissions, all of which have diurnal cycles (Fig. 2). This will result in a unique photochemical environment that varies as a function of time of day, day or week, and season. As such, understanding this changing environment and the implications for surface air quality and human health are not well served by satellite sensors in sun-synchronous orbits^116^. To address the combined air quality-human health challenge requires observations that can at least cover contiguous areas of 5 × 5 km^2^ (small cities) to 25 × 25 km^2^ (megacities) with a horizontal resolution that is aligned to individual streets (100 s m) throughout the day, complementing data that is beginning to become available from geostationary platforms^13,14,16^. The argument for installing an air quality-focused instrument on the ISS is shared with biogenic volatile organic compounds and the subsequent atmospheric chemistry.

#### Greenhouse gases

**Fig. 3:**
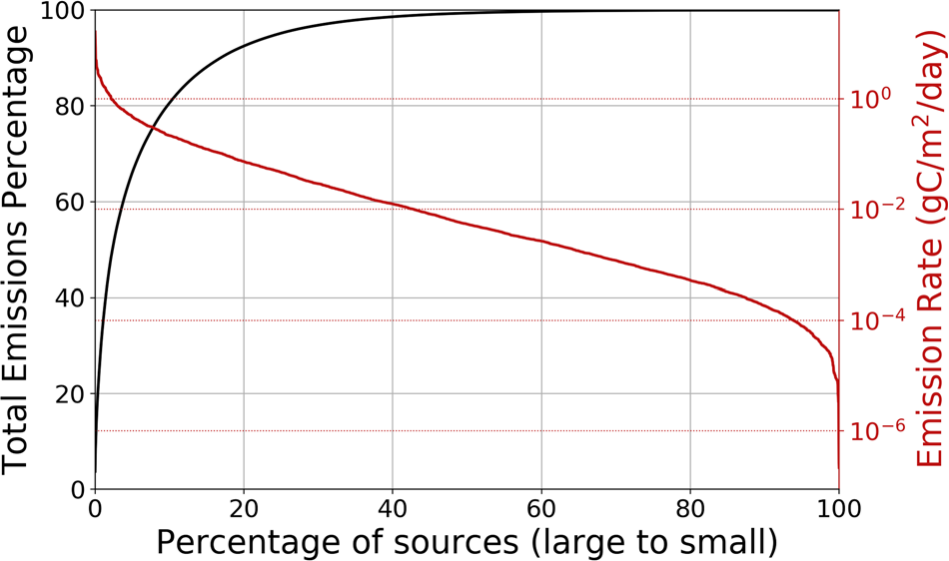
Inventory distribution of anthropogenic emissions of CO_2_ over the tropics in 2019 (refs. ^117,118^) as a function of total emissions. Figure drawn by D.F.P.

Figure 3 shows the annual tropical distribution of non-aviation, terrestrial anthropogenic CO_2_ emissions from the ODIAC inventory in 2019 (refs. ^117,118^), including point and non-point sources, cement production and gas flaring. Growth in international trade has increased dramatically in the tropics, with trade among developing countries doubling between 2004 and 2011, with implications for CO_2_ emissions particularly in the tropics^119^. Tropical emissions have a heavy-tail distribution so that a small number of large sources disproportionately influence the tropical emission total of 1.8 PgC. For example, 20% of the largest emissions correspond to more than 90% of all emissions. The larger emission hotspots can already be observed using existing low Earth orbiting satellites either directly as CO_2_^120–123^ or methane^124^, or via sensing tropospheric NO_2_^121,125–129^. A recent study combined satellite observations of CO and CO_2_ to improve source attribution across cities^130^. Hyperspectral sensors operating at ground resolutions of a few ten metres have been able to observe emission plumes of CO_2_ and methane from large coal-fired power-plants^131^ and from major leakages in the oil and gas sector^132,133^.

OCO-3 on the ISS (Table 1) has a SAMs observing mode that allows the instrument to map out CO_2_ columns over a limited geographical domain^76,130^. These limited domains have to date been almost exclusively over cities and calibration targets, but the flexibility enables the instrument to react to emerging scenarios, e.g., volcanic eruptions. This flexibility is enabled by an agile pointing mirror assembly that allows the instrument to collect off-nadir measurements^55^. A similar pointing system could be applied to other future instruments. If such a system were linked to an intelligent sampling system that included, for example, near-time cloud coverage information, a greater number of clear-sky scenes could be sampled, resulting in improved urban flux estimates of CO_2_ and methane. Combining fine ground-resolution of a few tens of metres with moderate spectral resolution^48,134^, the next generation sensor concepts promise to enable facility-scale carbon monitoring by quantifying CO_2_ and methane emissions not just for the largest power-plants and catastrophic methane leakages but also for smaller facilities representing a large fraction of the emission total. Active remote sensing systems that use a laser tuned to the centre (and to one side) of a CO_2_ absorption line would also provide measurement coverage during the daytime and nighttime and, more importantly, observe through broken clouds and to cloud tops. There are variety of lidar mission concepts for CO_2_ and methane that are in different stages of development^135,136^, which would also help improve understanding of regional, and potentially local, carbon budgets^137,138^. Demonstrating these concepts on the ISS would be an efficient way to mature the technologies while delivering urgently needed observation capabilities on anthropogenic emissions in the locations where population growth is fastest, although we acknowledge active systems will be more sensitive to platform stability.

## Underpinning the efficacy of emerging carbon markets

There is an urgent need to identify sustainable decarbonisation pathways for individual industries so they can meet net-zero emission targets by 2050. For many companies this will be a challenge without using some form of voluntary carbon offsetting scheme. Maintaining and expanding natural ecosystems over the tropics represents an example of a nature-based solution that could potentially deliver the necessary offset on decadal scales. But how do we ensure conservation and timber forests, and the associated ecosystems, truly deliver the necessary uptake in carbon? Offsetting schemes are typically informed by inventory estimates, but inferring carbon uptake from satellite observations of atmospheric CO_2_ is now a viable approach, complementing estimates inferred from above-ground biomass and from inventories^139^. Satellite observations of atmospheric CO_2_ could also deliver GHG emission estimates to help the industry sector to quantify their emissions and to help regulators and public bodies understand what can be measured.

Transparency is essential for any effective voluntary carbon offsetting market. No measurements are perfect and therefore should always be accompanied by some information on uncertainty that provides confidence bounds. If these uncertainties are provided then there is a wealth of Earth observation data that could be brought to bear on this challenge of monitoring carbon uptake, particularly over the tropics where there are very few alternative measurements, including estimates of above-ground biomass inferred from lidar and radar wavelengths^140–142^ and fluxes of CO_2_ inferred from atmospheric measurements of CO_2_^30,143,144^. Adoption of these data into the emerging market requires dialogue between the regulators and the science community to improve access to emerging scientific findings, infrastructure, and quality-assured datasets.

## Outlook and summary

At the time of writing the ISS is due to be phased out in 2030 when it will be subject to a controlled de-orbiting procedure leading to it being burned up in the Earth’s atmosphere somewhere over the South Pacific Ocean near Point Nemo, the oceanic pole of inaccessibility. The ISS is the ninth habitable space station (after Salyut 1, Skylab, Salyut 3–7, and Mir) and there remains extensive international interest in developing and maintaining space stations. The Chinese launched the Tiangong space station in April 2021, as part of the China Manned Space Program and building on Tiangong-1 and Tiangong-2. At the end of 2021, NASA commissioned three designs of space stations to ensure a transition from the ISS to a US-led low-Earth platform that will be available to the US government and to the private sector. ESA is also preparing a similar post-ISS low-Earth platform, currently called the SciHab (Science and Habitation) project. Neither the US or European platforms have identified Earth observation as a potential application, instead using the vantage point to support the exploration of the Moon and Mars. We argue that integrating an Earth observing capability on future space stations, unimpeded by the station structure and less affected by other space station operations would greatly improve our ability to observe changes in the Earth system, including the carbon cycle and air pollution.

There are also solely commercial space station concepts being developed. Axiom Space is due to launch Axiom Hab One in 2024, including crew quarters and space for research and manufacturing. This capability will be expanded with the subsequent launches of modules that include extended crew space, research, and manufacturing facilities. Orbital Reef is another commercial space station, led by Blue Origin and Sierra Space, that is due to be in orbit before 2030. They plan to build a station almost as big as the ISS, capable of supporting ten people, including research and development facilities. Their business plan includes the capability of installing third-party modules to the station infrastructure. This functionality goes far beyond the commercial opportunities available on the ISS, e.g., the Airbus Bartolomeo platform on the European Columbus module.

Future space stations may be placed in a range of low-inclination orbits depending on launch site latitude: it is cheapest (both financially and in terms of energy) to launch into an orbit with the same inclination as the launch site latitude. For the ISS the 51.6^o^ orbital inclination was driven by the need to support Russian launches from Baikonur. A US-only station may prefer a lower inclination orbit to better match the latitude of major US launch sites e.g., Cape Canaveral at ~28°N. This is especially true if the station is primarily being used as a deep space gateway rather than for Earth-related science, as minimising launch costs will be a primary driver for orbit selection. These lower inclination orbits would spend more time over the tropics, further improving coverage and increasing the number of cloud-free scenes recorded by onboard Earth observation instruments.

Tropical ecosystems are the subject of multifarious impacts driven by local to global societal and economic demands. Changes in land use to support, for example, agriculture and timber production and the exploration of rare earth minerals, driven by our growing use of semiconductors, has far-reaching implications for GHG emissions, biodiversity and human health^145–150^. Manned space stations provide an important vantage point for studying rapid changes in the tropics. They represent an environment for testing new, rapid-development Earth observing technologies that might eventually be launched on purpose-built satellites. They should also be considered as Earth observing platforms in their own right.

A key programmatic challenge to installing Earth observing instruments on any space station is the competition for time and space from other science experiments. Instrument time on the ISS is often limited to the shortest period that will demonstrate a measurement concept, which can be several years for Earth observing instruments. Looking forward in time, it is possible that the advent of commercial space stations, such as those described above, will help ease the competition for a suitable Earth-facing berth. Programmatic difficulties aside, if all new space stations supported a coordinated Earth measurement programme, taking advantage of their different orbital inclinations, we could establish a virtual tropical constellation of Earth observing platforms. These measurements would complement observations collected in sun-synchronous orbits and lead to a step-change in the number of clear-sky observations available to study the tropical carbon cycle and air pollution.

### Reporting summary

Further information on research design is available in the Nature Research Reporting Summary linked to this article.

## Supplementary information

Reporting Summary
